# Targeting Biological Aging: A New Paradigm for 21st Century Medicine

**DOI:** 10.1093/geroni/igab046.1828

**Published:** 2021-12-17

**Authors:** Matt Kaeberleien

**Affiliations:** University of Washington, North Bend, Washington, United States

## Abstract

Biological age is the greatest risk factor for nearly every major cause of death and disability, including COVID-19. Yet, traditional biomedical research and clinical approaches have focused on waiting until people are sick and treating individual diseases one at a time. Attempts to “cure” age-related diseases have proven unsuccessful, and the impact of “disease-first” approaches continue to be incremental. Recent advances in understanding them mechanisms linking biological aging to disease, or geroscience, have identified interventions that directly target the molecular hallmarks of aging. Unlike disease-specific approaches, such interventions have the potential to prevent multiple diseases of aging simultaneously, thereby greatly enhancing healthspan and lifespan for most individuals. Here I will provide an overview of translational geroscience, which I believe will become the paradigm for the practice of medicine in the 21st century. I will also discuss recent work with one such intervention, the drug rapamycin, and our efforts to eventually delay or reverse biological aging in companion dogs and people.

